# Complete genome sequence of “*Candidatus* Phytoplasma cynodontis” GY2015, a plant pathogen associated with Bermuda grass white leaf disease in Taiwan

**DOI:** 10.1128/MRA.00457-23

**Published:** 2023-09-15

**Authors:** Shu-Ting Cho, Ai-Ping Chen, Shu-Jen Chou, Xiao-Hua Yan, Chan-Pin Lin, Chih-Horng Kuo

**Affiliations:** 1 Institute of Plant and Microbial Biology, Academia Sinica, Taipei, Taiwan; 2 Department of Plant Pathology and Microbiology, National Taiwan University, Taipei, Taiwan; University of Maryland School of Medicine, Baltimore, Maryland, USA

**Keywords:** phytoplasma, genome, Mollicutes, pathogen, plant pathogens

## Abstract

The complete genome sequence of “*Candidatus* Phytoplasma cynodontis” strain GY2015, which consists of one 498,922-bp circular chromosome, is presented in this work. This uncultivated plant-pathogenic bacterium is associated with Bermuda grass white leaf disease in Taoyuan, Taiwan.

## ANNOUNCEMENT

An uncultivated bacterium, “*Candidatus* Phytoplasma cynodontis,” has been identified as the causal pathogen of Bermuda grass white leaf disease (1). This disease was first reported in Taiwan in 1972 (2) and later found in other Asian countries, Australia, Africa, and Europe (1). In addition to extensive chlorosis, the infection leads to stunting and death, causing economic damages in turf maintenance and worsening soil erosion. To facilitate the study of this pathogen, we conducted shotgun sequencing of a symptomatic plant collected in Guanyin District (Taoyuan City, Taiwan) in 2015 and assembled the complete genome sequence of strain GY2015 associated with this sample. All kits were used according to the manufacturer’s protocols, and all bioinformatics tools were used with the default settings unless stated otherwise.

Approximately 0.5 g of leaves was collected for DNA extraction using Wizard Genomic DNA Purification Kit (Promega), then used for both sequencing platforms. For Illumina sequencing, the library was prepared using KAPA LTP Library Preparation Kit (Roche) with a targeted insert size of 550 bp and sequenced on a MiSeq using Reagent Kit v3 (600 cycles) to generate 15.3 Gb of paired-end reads, then trimmed with a Q20 cutoff and filtered using a 200-bp cutoff. For Oxford Nanopore Technologies (ONTs) sequencing, the library was prepared using ONT Ligation Sequencing Kit (SQK-LSK109) without shearing and without size selection, then sequenced on a MinION (R9.4.1; FLO-MIN106). Basecalling was performed using Guppy v3.6.1 with the high-accuracy mode, which produced 546,782 reads (N50: 32.2 kb; combined: 11.7 Gb).

The first *de novo* assembly used only the Illumina reads for Velvet v1.2.10 (3). Putative phytoplasma contigs were identified by BLASTX (4) searches against phytoplasma proteins available in the NCBI nr database (5). All raw reads were mapped to these contigs using BWA v0.7.17 (6) and Minimap2 v2.15 (7). The Illumina reads with an alignment score >30 and the ONT reads with a score >500 were extracted for hybrid assembly using Unicycler v0.4.9b (8), which produced one circular chromosomal contig. This contig was polished iteratively using the Illumina reads based on BWA mapping and SAMtools v1.9 (9) checks. The procedure of gene prediction and annotation was based on that described in our previous work (10, 11). For gene prediction, RNAmmer v1.2 (12), tRNAscan-SE v1.3.1 (13), and Prodigal v2.6.3 (14) were used. The annotation was based on the homologs in other phytoplasma genomes available from GenBank (5) as identified by OrthoMCL v1.3 (15), followed by manual curation based on BlastKOALA v.2.2 (16). Putative-secreted proteins were identified using SignalP v5.0 (17) and filtered by TMHMM v2.0 (18).

The assembly consists of one 498,922 bp circular chromosome with 21.1% G + C content, which was rotated to have *dnaA* as the first gene. There were 1.0 million Illumina reads mapped to the phytoplasma genome, providing 597.5-fold coverage. For ONT reads, 2,256 reads totaling 56.4 Mb (N50: 33.9 kb) provided 113.1-fold coverage. The annotation contains 6 rRNA genes, 23 tRNA genes, 413 protein-coding genes, and 4 pseudogenes. No plasmid was found.

## Data Availability

This genome project has been deposited in NCBI under the accession number PRJNA318828. The metadata have been provided under the accession number SAMN04868714. The raw reads have been deposited at the NCBI Sequence Read Archive under the accession numbers SRX20434751 and SRX20434752. The genome sequence has been deposited at GenBank under the accession number CP126225.
